# International survey on complications of religious fasting after metabolic and bariatric surgery

**DOI:** 10.1038/s41598-023-47673-w

**Published:** 2023-11-18

**Authors:** Mohammad Kermansaravi, Farah A. Husain, Ahmad Bashir, Rohollah Valizadeh, Syed Imran Abbas, Tarek Abouzeid, Masoud Amini, Amir Hossein Davarpanah Jazi, Mohamad Hayssam Elfawal, Waleed Gado, Tikfu Gee, Tamer A. A. M. Habeeb, Bader Al Hadhrami, Atif Inam, Nader Moein Vaziri, Somayyeh Mokhber, Hazem Al-momani, Taryel Omerov, Abdolreza Pazouki, Alireza Rezapanah, Masoud Rezvani, Majid Sadat Mansouri, Alaa M. Sewefy, Halit Eren Taskin, Tahir Yunus, Radwan Kassir, Abdelrahman Nimeri

**Affiliations:** 1 Division of Minimally Invasive and Bariatric Surgery, Department of Surgery, Minimally Invasive Surgery Research Center, Rasool-E Akram Hospital, School of Medicine, Iran University of Medical Sciences, Tehran, Iran; 2 Department of Surgery, Banner University of Arizona-Phoenix, Phoenix, USA; 3 Minimally Invasive and Bariatric Surgery, Gastrointestinal Bariatric and Metabolic Center (GBMC)-Jordan Hospital, Amman, Jordan; 4 Urmia University of Medical Sciences, Urmia, Iran; 5 Iranian Hospital, Alwasl Road, Dubai, United Arab Emirates; 6 Department of Surgery, Ain-Shams University, Cairo, Egypt; 7 Laparoscopy Research Center, School of Medicine, Shiraz University of Medical Sciences, Shiraz, Iran; 8 Minimally Invasive Surgery Research Center, Isfahan University of Medical Sciences, Isfahan, Iran; 9 Makassed General Hospital, Beirut, Lebanon; 10 Endocrine and Bariatric Surgery Unit, Mansoura University, Mansoura, Egypt; 11 Department of Medical Science, School of Healthcare and Medical Sciences, Sunway University Jalan University, Selangor Darul Ehsan, Bandar Sunway, Petaling Jaya, Malaysia; 12 Department of General Surgery, Faculty of Medicine, Zagazig University, Zagazig, Egypt; 13 Royal Hospital, Muscat, Oman; 14 In Charge Metabolic, Thoracic and General Surgery Unit III, Department of General Surgery, Pakistan Institute of Medical Sciences, Islamabad, Pakistan; 15 Minimally Invasive Surgery Research Center, Iran University of Medical Sciences, Tehran, Iran; 16 NMC Royal Hospital, Abu Dhabi, United Arab Emirates; 17 First Surgical Disease Department, Azerbaijan Medical University, Baku, Azerbaijan; 18 Department of Surgery, Mashhad University of Medical Sciences, Mashhad, Iran; 19 Inova Fair Oaks Hospital, COE Bariatric Center, 14904 Jefferson Davis Hwy, Suite 205, Woodbridge, VA 22191 USA; 20 Pars Private Hospital, Tehran, Iran; 21 Department of Surgery, Minia University Hospital, Minia, Egypt; 22 Cerrahpasa Medical Faculty, Department of Surgery, Istanbul University Cerrahpasa, Istanbul, Turkey; 23 Evercare Hospital, Lahore, Pakistan; 24 Department of Digestive Surgery, Centre Hospitalier Universitaire Félix Guyon, St Denis de La Réunion, France; 25 Department of Surgery, Center for Metabolic and Bariatric Surgery, Brigham and Women’s Hospital, Harvard Medical School, Boston, MA USA

**Keywords:** Diseases, Gastroenterology, Medical research

## Abstract

Religious fasting in Ramadan the 9th month of the lunar year is one of five pillars in Islam and is practiced for a full month every year. There may be risks with fasting in patients with a history of metabolic/bariatric surgery (MBS). There is little published evidence on the possible complications during fasting and needs stronger recommendations and guidance to minimize them. An international survey was sent to surgeons to study the types of complications occurring during religious fasting in patients with history of MBS to evaluate the risk factors to manage and prepare more evidence-based recommendations. In total, 21 centers from 11 countries participated in this survey and reported a total of 132 patients with complications occurring during religious fasting after MBS. The mean age of patients with complications was 36.65 ± 3.48 years and mean BMI was 43.12 ± 6.86 kg/m^2^. Mean timing of complication occurring during fasting after MBS was 14.18 months. The most common complications were upper GI (gastrointestinal) symptoms including [gastroesophageal reflux disease (GERD), abdominal pain, and dyspepsia], marginal ulcers and dumping syndrome in 24% (32/132), 8.3% (11/132) and 23% (31/132) patients respectively. Surgical management was necessary in 4.5% of patients presenting with complications (6/132) patients due to perforated marginal or peptic ulcer in Single Anastomosis Duodenoileostomy with Sleeve gastrectomy (SADI-S), one anastomosis gastric bypass (OAGB) and sleeve gastrectomy (SG), obstruction at Jejunojenostomy after Roux-en-Y gastric bypass (RYGB) (1/6) and acute cholecystitis (1/6). Patients after MBS should be advised about the risks while fasting including abdominal pain, dehydration, and peptic ulcer disease exacerbation, and a thorough review of their medications is warranted to minimize complications.

## Introduction

Religious fasting is practiced in many religions. For Muslim patients, religious fasting in Ramadan the 9^th^ month of the lunar year is one of five pillars in Islam and is practiced for a full month in Ramadan every year. Fasting in Ramadan includes complete abstinence from eating and drinking from Suhoor (before dawn) to Iftar (after sunset) which may be more than 12 h in certain areas and the timing of the year. Notwithstanding the many metabolic benefits of fasting^1–3^, there may be risks in patients with history of metabolic/bariatric surgery (MBS). The risks of fasting after MBS may be higher in the presence of obesity related medical problems such as type 2 diabetes (T2DM), hypertension (HTN) especially when patients are using certain medications like insulin and diuretics. Also, the timing of fasting after MBS and the type of MBS may play a role in the occurrence of these complications. Despite the recently published experts' consensus^4^ and the American Society for Metabolic and Bariatric Surgery (ASMBS) position statement for religious fasting after MBS^5^ there is little evidence published on the possible complications during fasting and adequate recommendations and guidance to MBS teams on how to minimize complications due to fasting or how to manage them. Therefore, this international survey was conducted to examine the types of complications occurring during religious fasting in patients with history of MBS and evaluate the risk factors and provide more evidence-based recommendations.

## Methods

### Study design

An international survey was conducted to study the complications occurring during religious fasting in patients with a history of MBS. This survey was approved by the ethical committee of Iran University of Medical Sciences (Approval ID: IR.IUMS.REC.1401.030). Well-known bariatric surgeons with experience offering MBS to Muslim patients were invited to participate in the survey. The data were collected from April 2022 to July 2022. We reported information about age, gender, BMI at fasting, medications, comorbidities, and type of MBS (SG-LDJB: Sleeve Gastrectomy with Loop Duodeno-Jejunal Bypass; SG: Sleeve Gastrectomy; SADI-S: Single Anastomosis Duodeno-Ileal Bypass with Sleeve gastrectomy; SASI: Single-Anastomosis Sleeve Ileal Bypass; SASJ: Single-Anastomosis Sleeve Jejunal Bypass; BPD-DS: Biliopancreatic Diversion with Duodenal Switch; OAGB: One Anastomosis Gastric Bypass; RYGB: Roux-en-Y Gastric Bypass), complications (abdominal pain/ Epigastric pain/dyspepsia, nausea/vomiting, dehydration needs IV fluid, syncope, dumping, GERD, marginal ulcer, kidney stone, GI Bleeding, perforated marginal or peptic ulcer, acute cholecystitis and obstruction) and their management (surgery, EGD, PPIs, avoid fasting, conservative, IV fluid, Tranexamic acid).

### Patient selection

All patients with previous history of MBS who had complications occurring during religious fasting in Ramadan months 2021 and 2022 were included.

### Data collection

Metabolic/bariatric surgeons were asked to report data including patient demographics, BMI at the time of fasting, onset of fasting for the first time after MBS, type of MBS, complications and their management. Details from different centers were received through a classified Excel data reporting sheet. Collaborators were required to obtain their local approval for entering patient data into an international survey as per their local guidance, despite the IRB approval of survey. Finally, all data were merged by a single author for analysis. The patients with incomplete data were excluded from the survey.

Primary outcome was complications occurring during fasting in patients with pervious history of MBS and secondary outcomes were type of MBS procedures, management strategies used to treat these complications.

### Statistical analysis

Continuous data are presented as mean, standard deviation, while dichotomous data are reported as frequency, percentage and figure through Excel and SPSS version 22.

### Ethical approval statement

All procedures performed in the study involving human participants were in accordance with the ethical standards of the institutional and/or national research committee and with the 1964 Helsinki declaration and its later amendments or comparable ethical standards.

### Informed consent statement

Informed consent was obtained from the participants included in the survey.

## Results

### Quantitative information of the studied patients

In total 21 centers from 11 countries participated in this survey. They reported a total of 132 patients with complications after MBS who had admission to the surgical department or emergency department, 83.33% were females, mean age was 36.65 ± 3.48 years and mean BMI was 43.12 ± 6.86 kg/m^2^ at the time the complications occurring during fasting. Mean onset of first time to fast after MBS was 6.22 months. While the mean "Timing of complication occurring during fasting after MBS" was 14.18 months. On average, the fasting period per day was 12.70 h (time between Suhoor and Iftar). Of the patients with complications 11.3% were smokers (14/132) (Table 1).

**Table 1:** Quantitative information of the patients.

Variable	Min	Max	Mean	SD
Age (year)	17	65	36.65	3.48
BMI (kg/m^2^)	20.7	52.2	43.12	6.86
Onset of the first time to fast in (month)	1	24	6.22	5.10
Timing of complication occuring during fasting after bariatric/metabolic surgery (month)	1	77	14.18	8.34
Fasting period per day (time between Suhoor and Iftar) (hours)	7	18	12.70	3.17

*Min* minimum, *Max* maximum, *SD* standard deviation.

**Figure 1:**
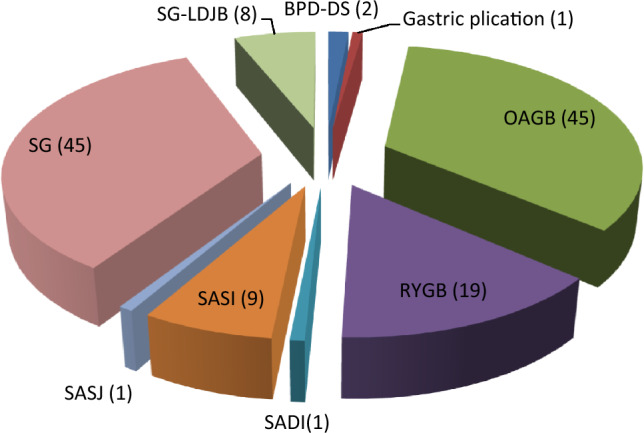
Types of surgery. *SG-LDJB* sleeve gastrectomy with loop duodeno-jejunal bypass, *SG* sleeve gastrectomy, *SADI-S* single anastomosis duodeno-ileal bypass with sleeve gastrectomy, *SASI* single-anastomosis sleeve ileal bypass, *SASJ* single-anastomosis sleeve jejunal bypass, *BPD-DS* biliopancreatic diversion with duodenal switch, *OAGB* one anastomosis gastric bypass, *RYGB* Roux-en-Y gastric bypass.

Regarding the type of MBS in patients with complications during fasting, sleeve gastrectomy (SG) (n = 46), one anastomosis gastric bypass (OAGB) (n = 45) and Roux-en-Y gastric bypass (RYGB) (n = 19) were the most frequent, respectively (Fig. 1).

### Current associated medical problems and medications in the studied patients

Obesity related medical problems for patients at the time of complications were reported included hypertension (HTN) 21% (28/132), type 2 diabetes mellitus (T2DM) 18.9% (25/132), dyslipidemia (DLP) 24.2% (32/132), GERD in 12.9% (17/132) patients, obstructive sleep apnea (OSA) in 6% (8/132) patients, and ischemic heart disease in one patient (0.8%). Most of patients were taking PPIs/H2blockers in 35% (41/132). Anti-hypertensive agents in 15% (18/132), lipid lowering agent in 10% (12/132), Levothyroxine in 8% (9/132), Allopurinol in 2% (3/132), NSAIDS in 2% (2/132), oral anti-diabetic/insulin in 12% (14/132), anticoagulant in 2% (2/132), antidepressant in 2% (3/132) and other medications (anti-hyperuricemic, urine alkalinizer, anti-migraine agent, corticosteroids, benzodiazepine) in 12% (14/132) were reported.

### Symptoms and complications in the studied patients

Esophagogastroduodenoscopy (EGD) was performed in 24% of patients with complications during fasting (32/132) with upper GI symptoms [gastroesophageal reflux disease (GERD), abdominal pain, and dyspepsia] and marginal ulcers in 8.3% of patients (11/132) most 8/11 after one anastomosis gastric bypass (OAGB) and 3/11 after (Roux-en-Y gastric bypass) RYGB]. Additionally, dumping syndrome occurred in 23.4% of patients (31/132), dehydration necessitating IV fluid in 18% of patients (24/132), nausea/vomiting in 25% of patients (33/132), syncope and kidney stone in 4% of patients (6/132), abdominal pain/epigastric pain/dyspepsia in 27% of patients (36/132). We report the complications in the Clavien-Dindo classification format in Table 2.

**Table 2:** Frequency of symptoms/complications (Clavien–Dindo classification) reported in the studied patients.

Clavien–Dindo classification	Symptom/complication	Frequency (%)
Grade I	Abdominal pain/epigastric pain/dyspepsia	36(27%)
Grade I	nausea/vomiting	33(25%)
Grade I	Dehydration needs IV fluid	25(19%)
Grade I	Syncope	6(4.5%)
Grade II	Dumping	31(23%)
Grade II	GERD	13(10%)
Grade II	Marginal ulcer	11(8%)
Grade II	Kidney stone	6(4.5%)
Grade II	GI bleeding	2(1.5%)
Grade IIIb	Perforated marginal or peptic ulcer	4(3%)
Grade IIIb	Acute cholecystitis	1(0.7%)
Grade IIIb	Obstruction (food bolus)	1
–	Others^a^	39

^a^Others: hypoglycemia/hyperglycemia, diabetic ketoacidosis, bloating, constipation, food poisoning, diarrhea, gout.

### Management approach in the studied patients

Regarding management of complications, most complications were managed utilizing conservative and supportive approaches including medical management in 36% of patients (48/132), dietary/lifestyle modification in 35.6% (47/132), rehydration (IV fluid) in 10.6% (14/132), avoiding fasting in 9% (12/132) and surgical management in 4.5% of patients [6/132] (4/6 patients due to perforated marginal ulcer after Single Anastomosis Duodenoileostomy [SADI-S],and OAGB or perforated peptic ulcer after Sleeve gastrectomy, 1/6 patients due to food bolus and obstruction at jejuno-jejunal anastomosis about three months after RYGB and laparoscopic cholecystectomy in 1/6 patients due to acute cholecystitis).

### Details of patients with complications

#### (a) Peptic ulcer complications (perforated marginal ulcer or peptic ulcer and GI bleeding)

Four patients developed perforated marginal ulcer or peptic ulcer after single anastomosis duodeno–ileal bypass with sleeve gastrectomy (SADI-S), and one anastomosis gastric bypass (OAGB) or perforated peptic ulcer after SG, of which 2/4 were current smokers, none used Aspirin/NSAIDs. The, the onset of Perforated marginal or peptic ulcer during fasting was more than 6 months in all four patients after MBS (6, 12, 12 and 16 months). Additionally, 11 patients developed marginal ulcer [three after RYGB (N = 3) and eight after OAGB] only two were current smoker. Only one patient with osteoarthritis was taking NSAIDS/Aspirin. Timing of MU during fasting after MBS varied from 6 to 37 months. Their BMI varied from 22.7 to 52.2 kg/m^2^. There were two patients with GI bleeding after SG 0/2 who were using Aspirin/NSAIDs or current smokers. (Table 3).

**Table 3:** Characteristics of patients with PPUD.

Variable	Age	Gender	BMI at fasting	Type of MBS	A	Smoking	NSAIDS/aspirin	B	Associated medical problems	Medications	Management approach
Perforated marginal or peptic ulcer	46	Female	35	SADI	6	No	No	17	DLP	PPIs/H2blocke	Surgery
	35	Male	37	SG	12	Yes	No	14	HTN	Anti-hypertensive + lipid lowering agent	Surgery
	37	Male	26	OAGB	16	Yes	No	14	–	–	Surgery
	38	Female	30	OAGB	12	No	No	14	–	–	Surgery
Marginal ulcer	57	Male	41	OAGB	9	Yes	No	16	DM + HTN + OSA	Oral anti-diabetic + anti-coagulant	EGD, PPIs, avoid fasting
	38	Female	29.4	RYGB	9	No	No	15			EGD, PPIs, avoid fasting
	44	Female	41	RYGB	7	No	No	14	DM + HTN	Insulin + lipid lowering agent + PPIs/H2blocke	PPIs
	56	Female	29	OAGB	11	No	No	9	IHD	Anti-coagulant	PPIs
	25	Female	31	OAGB	16	No	No	9			PPIs
	49	Male	36	OAGB	9	No	No	8.5			PPIs
	56	Female	34	OAGB	10	No	Yes	8	Osteoarthritis	NSAIDS/aspirin	PPIs
	43	Male	52.2	OAGB	77	Yes	No	15		PPIs/H2blocke	EGD, PPIs
	46	Female	22.66	RYGB	37	No	No	15		PPIs/H2blocke	EGD, PPIs
	41	Female	27.59	OAGB	22	No	No	15		PPIs/H2blocke	EGD, PPIs
	36	Female	35.49	OAGB	6	No	No	15			EGD, PPIs
GI bleeding	33	Female	32.4	SG	10	No	No	16	–	–	Conservative, IV fluid
	26	Female	39	SG	12	No	No	15	HTN	Anti-hypertensive	Conservative, IV fluid, Tranexamic acid

A: Timing of complication during fasting after bariatric/metabolic surgery (months).

B: Fasting time (time between Suhoor and Iftar) (hours).

#### (b) Patients who needed IV fluid due to dehydration

Twenty-five patients (18.9%) needed IV fluids for dehydration. Mean age was 38.76 ± 9.89 years while BMI was 30.27 ± 5.44 kg/m^2^. The mean timing of dehydration requiring IV fluid therapy was 16.52 ± 14.21 months and length of fasting (time between Iftar and Suhoor) in (hours) was 11 ± 4.38 h. Two patients (8%) were smokers. Type of MBS is detailed below (Table 5). Most patients with dehydration needed IV fluid around 9 h after starting their fast (range 7–24 h (Table 4).

**Table 4:** List of the patients who need IV fluid due to dehydration.

Age	Gender	BMI at fasting	Type of surgery	A	Smoking	B	Associated medical problems	Medication
38	Male	30	SASI	36	Yes	7	–	–
17	Male	25.5	SASI	8	No	9	HTN + DLP + NAFLD	–
35	Male	27	SG-LDJB	7	No	8	HTN + NAFLD	–
41	Female	28.3	SASI	26	No	24	–	–
34	Female	29.64	OAGB	7	No	15	–	Clonazepam
36	Female	26.04	OAGB	10	No	15	–	PPIs/H2blocke
36	Female	28.42	OAGB	26	No	15	–	–
37	Female	26	SASI	18	No	7	–	–
54	Female	28.3	OAGB	16	No	7	–	–
52	Female	22	RYGB	18	No	7	–	–
28	Female	34.8	SG	15	No	16	–	–
43	Male	30	SG	1	Yes	14	DLP	Lipid lowering agent
37	Female	32	SG	5	No	9	T2DM + HTN	–
22	Female	46.1	SG	1	No	9	–	PPIs/H2blocke
58	Female	38.3	OAGB	8	No	9	Osteoarthritis	–
34	Female	28.5	SG-LDJB	6	No	9	–	Levothyroxine
34	Female	38	RYGB	8	No	11	DLP	–
48	Female	25	SG-LDJB	11	No	7	NAFLD	–
30	Female	28.5	BPD-DS	60	No	15	Lymphoma	–
47	Male	26.5	BPD-DS	48	No	15	T2DM	–
38	Female	24	SG-LDJB	17	No	15	DLP + NAFLD, migraine headache	–
48	Female	28.7	SG	25	No	7	–	–
45	Female	34.2	SG	18	No	7	–	–
28	Female	34	SG	6	No	8	GERD	PPIs/H2blocke
49	Male	37	SG	12	No	14	HTN + GERD	Anti-hypertensive + PPIs/H2blocke

A: Timing of complication during fasting after bariatric/metabolic surgery (months).

B: Fasting time (hours).

*HTN* hypertension, *DLP* dyslipidemia, *T2DM* type 2 diabetes mellitus, *NAFLD* non-alcoholic fatty liver disease, *GERD* gastroesophageal reflux disease.

#### (c) Details of patients with kidney stones

Four patient developed kidney stones during fasting and that had history of sleeve gastrectomy with duodeno-jejunal bypass (SG + DJB), OAGB and SG after fasting for 9, 15, 16 and 15 h (time period between Suhoor and Iftar). Of the four patients with kidney stones, two patients were smokers, and taking PPIs, Details are in Table 5.

**Table 5:** Characteristics of patients with kidney stone.

Age	Gender	BMI at fasting	Type of MBS	A	Smoking	B	Associated medical problems	Medications
33	Female	23	SG + LDJB	18	No	9	DLP + NAFLD	PPIs/H2blocke
28	Male	29	OAGB	9	Yes	15	–	NSAIDS/aspirin
32	Female	28	SG	11	Yes	16	GERD	PPIs/H2blocke
42	Male	27.69	OAGB	20	No	15	GERD	–

A: Timing of complication during fasting after bariatric/metabolic (months).

B: Fasting time (hours).

#### (d) Details of patients with glucose disorder

Two patients developed hypoglycemia and hyperglycemia, previously underwent SG and OAGB with 16 and 9 h fasting time in hours and the patient with hypoglycemia was a smoker. Details are below (Table 6).

**Table 6:** Characteristics of the patients with hypoglycemia and hypoglycemia.

Case with	Age	Gender	BMI at fasting	Type of MBS	A	Smoking	B	Associated medical problems	Medication
Hypoglycemia	51	Female	35.2	SG	12	Yes	16	None	None
Hyperglycemia	56	Female	43.6	OAGB	7	No	9	T2DM	Oral anti-diabetic
Diabetic Ketoacidosis	26	Female	39	SG	12	No	14	T2DM	Insulin

A: Timing of complication during fasting after bariatric/metabolic (months).

B: Fasting time (hours).

## Discussion

Religious fasting is a tenet in many religions around the world and the ability to fast after MBS is an important discussion point for patients considering MBS. Religious fasting during the month of Ramadan is an extremely important pillar to Muslims. It remains unclear when is it safe for patients to start fasting in Ramadan after MBS and what are the risks of fasting after MBS. Two recent studies made recommendations on religious fasting based on expert consensus^4^ and a literature review^5^. In both studies, collaboration between the patient and the bariatric multidisciplinary team and practicing shared decision-making were at the forefront of recommendations. This is the first survey to look at complications following MBS when patients resume fasting in Ramadan which involves more than 10 h of fasting on average from food and water.

This survey reviewed patients with history of MBS who were fasting during the month of Ramadan in the 2021 or 2022 and were subsequently seen for a complication related to MBS. Sleeve gastrectomy and OAGB were the predominant procedures performed in patients with complications while fasting, followed by RYGB which is indicative of the international pool of surgeons contributing to the data and is consistent with previous report of MBS in the Middle East region^6^. On average, patients resumed fasting slightly over a year after MBS and participated in a fast from dusk to dawn that was approximately 13 h long. Fasting in Ramadan is unique in that participants refrain from drinking and eating during the fasting period. The fast includes not taking medications during the daytime, and thus may alter how patients are taking medication or if they are taking medications.

Perforated or bleeding marginal or peptic ulcers were the most common major surgical complications, and they occurred after SADI-S, OAGB, and SG. Additionally marginal ulcers after OAGB or RYGB or peptic ulcers in the duodenum after SG were frequently reported during Ramadan fasting^7^. When monitoring the healing of marginal or peptic ulcers during Ramadan or after fasting, PPIs remain the mainstay of treatment and are successful 90% of the time as a management strategy^8^. The etiology of marginal or peptic ulcers in patients with previous MBS during fasting remains unclear, but based on previous literature, regular use of PPI may be considered for prevention and treatment during fasting. Most patients with marginal or peptic ulcers had OAGB, but more OAGB was performed versus RYGB in this population. According to the findings summarized in Table 3, only one patient on PPIs had perforated peptic ulcer disease (PPUD), and other patients on PPIs had no occurrence of PPUD, so it may be advisable to instruct patients to take PPI throughout any periods of fasting.

The most avoidable operative intervention was a food bolus leading to obstruction of the jejunojejunostomy after RYGB but this occurred in only one patient. Reviewing postoperative dietary recommendations and mindful eating prior to fasting may be important to remind patients to avoid eating too quickly and not chewing their food well upon breaking fasts.

Dehydration was reported in 25 patients and kidney stones, which are likely closely related to dehydration, in six patients. Patients with dehydration required IV hydration and they had different types of MBS, so one procedure was not more prone to dehydration. Only 12 patients received recommendations to avoid fasting after being seen and treated. With this level of intervention required, it may be prudent to recommend short-term avoidance of fasting in patients who require IV fluids or develop kidney stones after MBS. Hydration with up to 2L of water is recommended for kidney stone prevention after bariatric surgery^9^. Given the rates of dehydration, the care team can strategize with patients how and when to hydrate and create a schedule for drinking fluids before starting the fast and after breaking the fast in the evening.

Abdominal pain was the most common symptom in patients with history of MBS during fasting. It could not be discerned if this was chronic or acute in this survey. There are several reasons for abdominal pain during fasting, including the risk of overeating when breaking the fast and eating foods high in fats and sugars that can lead to dumping syndrome^9^. Patients need re-education on smoking cessation, as many patients with complications after MBS were actively smoking. In addition, education and reinforcement of vitamin supplementation may help prevent kidney oxalate stone that can be best treated by taking calcium supplementation. Calcium is often broken down and taken over three to four times per day, which is not as feasible during a full daytime fast. It would be prudent to screen patients for a history of kidney stones before they begin fasting, as previous history can increase quadruple the risk of having stones later^9^. Since kidney stone recurrence is one of the complications, patients with this history may need to be advised to avoid fasting or given strict hydration instructions.

Glycemic control during fasting did not appear to be a common issue, and in the 3 patients affected by blood sugar variances, there were no obvious patterns. It would be important to track food logs in this group of patients and understand intake during eating hours and medication compliance. One patient was on insulin, but it is unclear if the patient was taking insulin while fasting. Diabetic patients on insulin, should be advised to consult with their endocrinologist before fasting if they had MBS^10^. Diabetic ketoacidosis (DKA) can be a life-threatening complication and should be prevented. Although the risk of DKA is very low in T2DM patients during Ramadan fasting^11^, the risk can be increased because of excessive reduction of insulin dose due to the fact that the patient is avoided from eating food during the fasting^10,12^. Another etiology of DKA is increasing of ketogenesis and gluconeogenesis as a result of excessively breakdown of glycogen due to insulin deficiency during fasting^13^.

A practical guideline about diabetes and Ramadan, recommend to self-monitoring of blood glucose by diabetic patients and break the fast in presence of blood glucose levels > 300mg/dl (16.6 mmol/L)^10,12^. Diabetic patients who had history of MBS are also advised to avoid sweets and high-sugar foods at Iftar to avoid DKA ^4^. A history of DKA within three months before Ramadan can be considered as high-risk patient and may recommend avoiding fasting^14^. Hyperglycemia was seen in a patient on oral medications, so again, medication compliance and consuming high carbohydrate foods after fasting may have contributed to this finding.

Religious fasting after MBS remains a reality that many of our patients encounter. Studies need to continue to explore adverse outcomes and if they can be avoided during periods of fasting. This survey contributes types of complications seen at an average of 14.2 months after MBS when resuming Ramadan fasting. However, further understanding of medication compliance, and diet details is needed. It would be helpful to have patients maintain diet logs to understand types of food being consumed, track amount of fluid intake for hydration, and when medications are taken. While this data gives some background about the risks of fasting, and areas to monitor in our patients, it does not define when it is safe for patients to resume fasting after MBS.

This survey despite being the largest collection of 132 patients with complications after fasting with history of MBS, the survey has several limitations. This was a survey and we were not able to determine the prevalence of these complications and differentiate if certain types of surgery are safer and offer less complication rates. In addition, it would be helpful to have vitamin levels and other lab studies to understand nutritional status when fasting is started. In addition the survey does not have a control group and may not answer the question of causality.

Finally, medical history is not provided, and some conditions may guide resumption of fasting vs avoidance. Ideally, prospectively collected database with preoperative factors, surgical details, and postoperative information would help clarify some of these limitations within our survey.

In summary, MBS is performed globally, and obesity is a global health issue and MBS may change common religious practices like fasting. For some patients, being unable to fast may make MBS untenable as an option and they need open counseling and advice. Patients should be advised about the risks of abdominal pain, dehydration, and marginal/peptic ulcer disease exacerbation while fasting, and review medications with their care team to garner advice about when and how to take medications. Work should be continued to identify how fasting can be done safely in the patient population, and in the meantime, patients should stay in close contact with their weight management team during periods of religious fasting.

Recommendations:

1. Prior to commencing religious fasting, it is advisable for patients to engage in consultations with their surgeons and multidisciplinary teams (MDT).
2. The regular utilization of proton pump inhibitors (PPIs) may be contemplated as a preventive and therapeutic measure for marginal ulceration (MU) during fasting.
3. After MBS, it is essential to provide patients with postoperative dietary recommendations and promote mindful eating habits before fasting. This serves as a reminder for patients to avoid rapid consumption and encourages thorough chewing when breaking their fasts.
4. To prevent the formation of kidney stones following MBS, it is recommended to maintain adequate hydration by consuming up to 2 L of water.
5. Collaborate with patients to develop strategies for effective hydration, including determining the appropriate timing and quantity of fluid intake before initiating the fast and after breaking it in the evening.
6. Patients necessitate re-education regarding smoking cessation, particularly as many individuals who experienced complications after MBS were actively smoking.
7. Diabetic patients who are on insulin therapy should be advised to consult their endocrinologists before embarking on fasting if they have undergone MBS.
8. Diabetic patients should engage in self-monitoring of their blood glucose levels and discontinue fasting if their readings exceed 300 mg/dl.
9. Patients with a history of diabetic ketoacidosis (DKA) within the three months preceding the Ramadan period may be classified as high-risk individuals, and it may be advisable for them to abstain from fasting.
10. Diabetic patients with a history of MB are also encouraged to refrain from consuming sweets and high-sugar foods during Iftar in order to prevent the DKA.

## Data Availability

The datasets used and/or analyzed during the current survey available from the corresponding author on reasonable request.
